# Healing of Post-Extraction Sockets Filled with Anorganic Bovine Bone and Covered with a Xenogeneic Collagen Matrix. Radiological 2D and 3D Results of a Pilot Study to Assess Dimensional Stability

**DOI:** 10.3390/ma14102473

**Published:** 2021-05-11

**Authors:** Tiziano Testori, Matteo Antonio Deflorian, Federico Mandelli, Giulia Attardo, Carlo Maiorana, Massimo Del Fabbro, Raffaele Vinci

**Affiliations:** 1 Dental Clinic, IRCCS Orthopedic Institute Galeazzi, 20161 Milan, Italy; tiziano.testori@unimi.it; 2 Department of Periodontics and Oral Medicine, School of Dentistry, The University of Michigan, Ann Arbor, MI 58259, USA; 3 Section of Implantology and Oral Rehabilitation, Dental Clinic, IRCCS Orthopedic Institute Galeazzi, 20161 Milan, Italy; deflon@hotmail.it; 4 Dental School, Vita-Salute University, IRCCS San Raffaele, 20132 Milan, Italy; federico.mandelli@gmail.com; 5 Center for Edentulism and Jawbone Atrophies, Fondazione IRCCS Cà Granda Ospedale Maggiore Policlinico, 20122 Milan, Italy; studio@attardoparrinelloluciano.191.it; 6 Department of Biomedical, Surgical and Dental Sciences, University of Milan, 20122 Milan, Italy; carlo.maiorana@unimi.it; 7 Dental Clinic, Fondazione IRCCS Cà Granda Ospedale Maggiore Policlinico, 20122 Milan, Italy; 8 Graduate school of oral surgery, Dental School, Vita-Salute University, 20132 Milan, Italy; vinci.raffaele@hsr.it; 9 Department of Dentistry, IRCCS San Raffaele, 20132 Milan, Italy

**Keywords:** extraction socket, socket seal, ridge preservation, 3D evaluation, implantology

## Abstract

Analysis of short-term results regarding dimensional stability of post-extraction sockets managed via a preservation protocol using deproteinized bovine bone matrix and a xenogeneic collagen matrix. Materials and methods Fifteen patients needing extraction of one single-rooted premolar tooth were treated in a pilot study. Five patients were treated in each centre. After tooth extraction, sockets were filled with anorganic bovine bone matrix and covered with a xenogeneic collagen matrix. Six months later, implants were placed. Dimensional changes in the treated sites were digitally evaluated using the best-fit superimposition of pre-and post-socket preservation models. Results After six months of healing, the vertical reduction of the grafted sites was 0.31 ± 0.24 mm (*p* < 0.001). Volumetric analysis of superimposed models showed an average palatal-lingual contraction of 0.33 ± 0.51 mm^3^ (*p* = 0.02). At the vestibular level, the average contraction was found to be 0.8 ± 0.3 mm^3^ (*p* < 0.001). Finally, the analysis of linear variations in the treated sites on a single sagittal section at the crystal level, and at 3 and 7 mm apically respect to the crest, both towards the vestibule and palate, generally showed more marked resorption at the crestal level compared to apical measurements. Conclusion: The clinical protocol herein employed for socket preservation showed a positive effect in preventing the physiological post-extraction remodeling.

## 1. Introduction

Following tooth extraction, remodeling of the hard and soft tissues is observed leading to a horizontal volume reduction in the alveolar crest, mainly involving the vestibular area with respect to palatal–lingual side [1,2,3]. Post-extraction morphological alteration has been analyzed in numerous studies. Araujo and Coll. in 2006 showed how the vestibular bone wall plays a fundamental role in the remodeling process [3]. Once the tooth is extracted, blood flow via periodontal ligament is undermined. In the case of a thin vestibular wall consisting only of cortical bone, blood flow coming from the periosteum is not sufficient to fully preserve this structure. The dimensional variations were analyzed both in animal and human models, using various methods ranging from the radiographic evaluation to clinical assessment of post-extraction sockets to analysis of pre-op and post-op scans of plaster models [4,5,6,7]. From a clinical standpoint, the average resorption of post-extraction sockets is around 50% of the vestibular–palatal–lingual width and mostly occurs within the first three months following tooth extraction [8,9]. Such volume loss may cause problems to dental implant placement [8,9]. Several surgical approaches have been proposed to maintain the volume of post-extraction sockets unaltered: placement of immediate post-extraction implants with biomaterial grafts in the peri-implant gap, or socket preservation techniques with or without utilizing covering membranes or soft tissue grafts. Nevertheless, these techniques are unable to completely avoid post-extraction volume loss [10,11]. The present, prospective clinical study aimed to assess clinically, radiographically and histomorphometrically a novel protocol for socket preservation using an organic bovine bone protected by a porcine collagen matrix. Preliminary clinical and histological results of this study have been published previously [12]. In this report, the radiographic results are presented, with special focus on digital evaluation.

## 2. Materials and Methods

**Figure 1:**
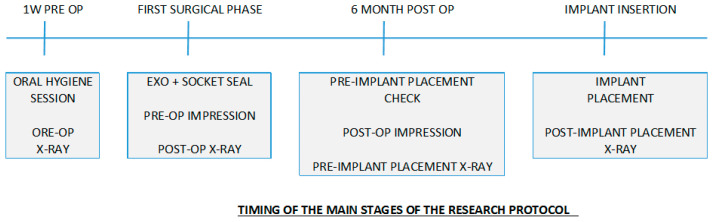
Timing of the main stages of the protocol during soft tissue analysis.

A prospective, multicenter clinical study was carried out at three centers of two universities in Milan, Italy (Università degli Studi di Milano, Università “Vita e Salute” San Raffaele). All patients received their treatment according to the principles enclosed in the World Medical Association Helsinki Declaration for biomedical research involving human subjects of 1975, as revised in 2000. All subjects gave their informed consent for inclusion before they participated in the study. The study was conducted in accordance with the Declaration of Helsinki, and the protocol was approved by the Institutional Review Board of the IRCSS Orthopedic Institute Galeazzi as part of a larger research project with exemption from ethical approval, and registered with the Prot. No. 75/2019 (Project Code: L2057). Patients’ selection criteria were the following. Inclusion Criteria: one or more compromised premolar teeth in need for extraction in the upper and/or lower jaws; Intact alveolar bone walls; Patients ASA-1 or ASA-2, able to undergo surgical treatment; Patients had to be able to understand and sign an informed consent form, and available to return periodically for scheduled control visits. Exclusion Criteria: general contraindication to oral surgery; immunosuppressed patients; uncontrolled diabetes; pregnancy or breast-feeding; presence of active periodontal disease, poor oral hygiene habits and motivation; known allergy to collagen; drug or alcohol addiction; psychiatric issues; presence of acute infection (e.g., abscess) or suppuration into or around the extraction socket. Before recruitment all the patients signed an informed consent form. Patients were also informed of alternative treatments. All included subjects had at least one extraction site, scheduled to receive an implant six months after extraction. However, only one tooth per patient was considered. In case a patient had more than one premolar treated, the tooth selection was made randomly. The study continued until one week after implant placement, i.e., six months and one week after tooth extraction (Figure 1).

A week before surgery the patients underwent a professional oral hygiene session whereby they were instructed on how to correctly perform a mouth rinse containing 0.12% Chlorhexidine for one minute twice a day. They were prescribed antibiotic therapy (1 g every 8 h) using Amoxicillin, starting the first day of surgery until six days after surgery. Before tooth extraction two photographs of the site (frontal and occlusal) were taken, a peri-apical radiograph was done using customized film holder, and a polyether (Impregum™ Penta™ Soft-3M, Pioltello, Milano, Italy) impression was made. The latter was developed subsequently, with extra-hard plaster of Class IV (GC FujiROCK EP, GC Corporation, Tokyo, Japan), to have a baseline volume reference. The tooth under investigation was colored black with a felt tip pen. Only intact post-extraction alveoli were considered suitable for the study. Immediately after tooth/teeth extraction, some clinical measurements such as alveolar crest width (vestibular–palatal–lingual dimension) were recorded, taken at the upper part of the crest, halfway from the extraction site in the mesio-distal direction. All clinical measurements were recorded by using a periodontal probe, and a periosteal elevator was used to expose a 1-mm portion of the lingual/palatal bone. The tip of the probe was positioned in such a way that it was possible to effect the measurements as coronal as possible. Post-extraction alveoli were filled with deproteinized, bovine bone granules of size 0.25–1 mm (Bio-Oss®, Geistlich Pharma AG, Wolhusen, Switzerland) up to the coronal margin of the alveolar bone compacting the biomaterial and preserving the granule size, i.e., avoiding crumbling. The biomaterial was covered with porcine collagen matrix (Mucograft® Seal, GeistlichPharma AG, Wolhunsen, Switzerland). After having measured the socket dimensions, the Mucograft Seal, which is a circular collagen matrix whose diameter is 8 mm, was reshaped to the right size utilizing an Iris scissor. The compact side (the thinner part) of the matrix faces outwards whereas the spongy side (with striations) faces the alveolus. The external borders of the matrix in contact with surrounding disepithelized soft tissues were sutured with single, interrupted stitches (PGA 6.0 o 7.0 STOMA, Emmingen-Liptingen, Germany) avoiding any tension. The sutures were removed three weeks later. The patient was recommended not to brush the surgery site for four weeks and to use a 0.12% Chlorhexidine mouthrinse, starting 24 h after the surgery. Four weeks after the intervention, the patient began using a special, extra-soft brush for the next four weeks after which began using a standard toothbrush. Six months following tooth extraction and socket preservation, and prior to implant placement, a new, polyether impression (Impregum™ Penta™ Soft-3M), was taken to be subsequently developed with extra-hard plaster class IV (GC FujiROCK EP) to compare with the initial 3-D images (for tooth extraction). During the implant placement surgery, a keratinized soft tissue sample was taken using a punch scalpel with a circular blade of 4 to 6 mm diameter, depending on site, and subsequently a bone biopsy was taken using a trephine with a 2.5 mm diameter. Both biopsies taken from each socket, in the same sites in which implants were planned, were preserved in 10% buffered formalin solution and then sent for histological and histomorphometric evaluation. During implant placement and subsequent carrying out of mucous tissue and bone biopsies the width of the alveolar crest (vestibular–palatal–lingual size) was evaluated again. The assessment of the volume variations in the crest was therefore obtained by comparing the clinical measurements made during the surgical procedures 1 and 2 and recorded on the Patient’s Form. One week after implant placement, during check-up, sutures were removed and photographs (frontal and occlusal) taken.

Radiological Assessment

Radiographic assessment of alveolar sites was carried out using a dedicated Rinn centering device with a resin holder so that the precise position could be repeated in various stages. Regarding the use of Mucograft Seal, alveolar size measurements were made on pre- and post-op radiographs at four stages: pre-extraction; post-socket coverage with soft tissue, pre-implant placement six months after socket coverage, post-implant placement. In particular, for each radiograph, the vertical size of the socket pre- and post-extraction was measured via software for the analysis and measurement of biomedical images [13].

Analysis of Models

**Figure 2:**
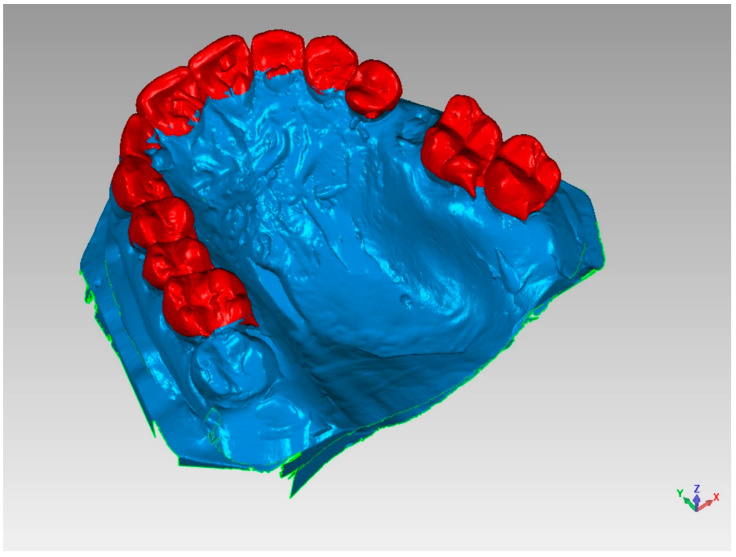
Optical scan of study models for the analysis of volumetric variations.

**Figure 3:**
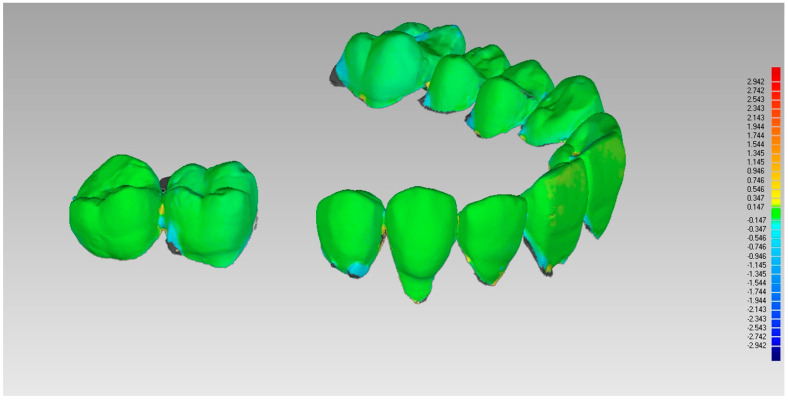
Superimposition of pre and post models based on the best fit of tooth surface.

Initially, an optical scan (scanner SmartOptics Activity 885, Bochum, Germany) of models 1 (pre-extraction) and 2 (pre-fixture, six months after extraction) was carried out with the creation of a relative mesh in.stl format (Figure 2). Then, model 2 was placed over model 1, directing the superimposition thanks to the best-fit principle of the dental or mucosal surfaces not subjected to intervention, i.e., areas that did not undergo volumetric variations between models 1 and 2 (Figure 3).

**Figure 4:**
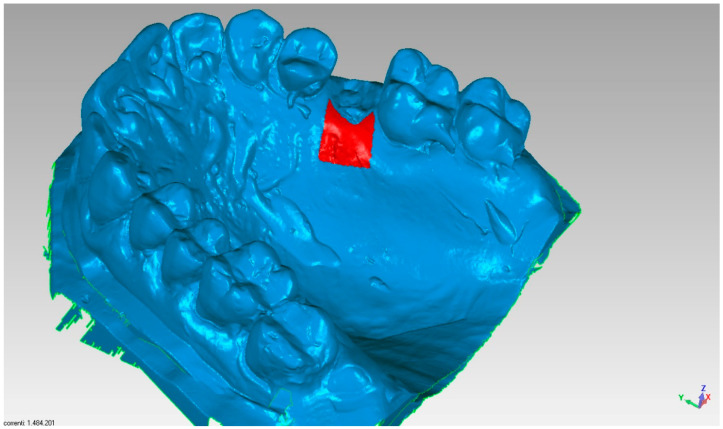
Area of analysis regarding palatal volume variations.

**Figure 5:**
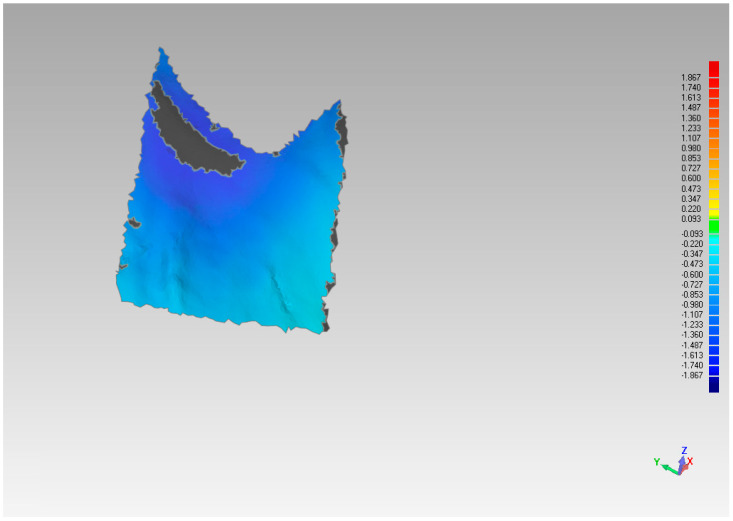
Analysis of palatal variation in the pre-implant placement model compared to pre-extraction model.

**Figure 6:**
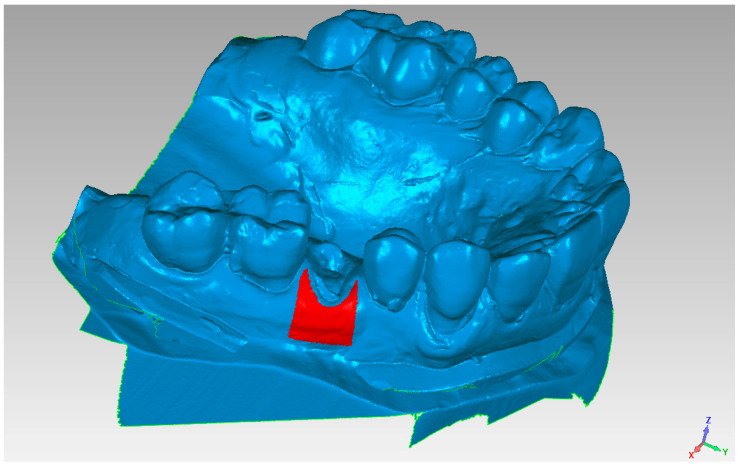
Area of analysis regarding vestibular volume variations.

**Figure 7:**
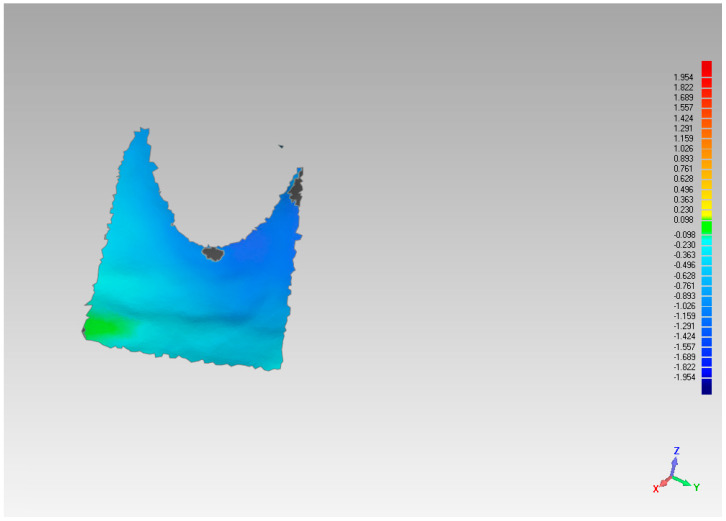
Analysis of vestibular variation in the pre-implant placement model compared to the pre-extraction model.

The assessment of the pre and post-extraction volume variation was carried out via software 3Diagnosys (3Diemme, Milan, Italy). In addition, an analysis of the single variation on a sagittal–palatal/lingual (Figure 4 and Figure 5) and vestibular section, (Figure 6 and Figure 7) was made.

**Figure 8:**
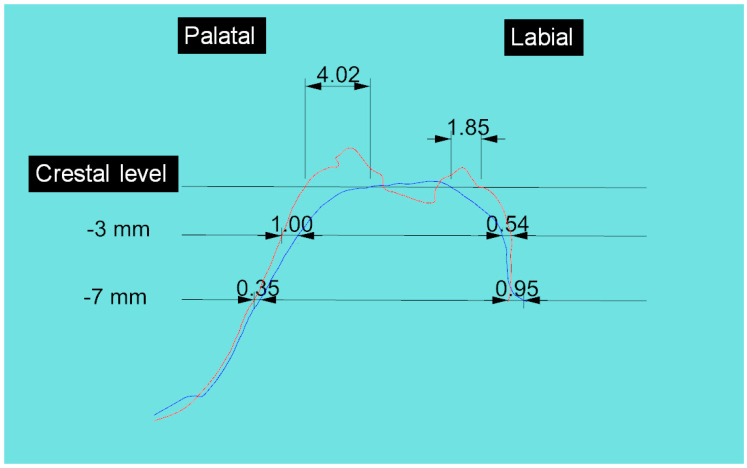
Sagittal superimposition of pre/post models with linear variation at the crestal level, at 3 mm and 7 mm from the crest.

To carry out the analysis, a line was first drawn in the vestibular to palatal/lingual direction on the upper part of the crest, midpoint in the mesio-distal direction of the alveolar site in order to obtain a single sagittal vestibular, and palatal/lingual section. These were used as reference points for further measurements in the superimposed model 2 over model 1. From these cross-sections, it was possible to measure quantitatively the variations and deviation in models 1 and 2 (Figure 8).

Pre and post-extraction measurements of the vestibular to lingual/palatal width (in the horizontal direction) were made at three levels: crestal level, 3 and 7 mm apical respect to the crest

## 3. Results

Fifteen patients were enrolled at three centers in Northern Italy (five patients per centre). The cases were treated by three clinicians (CM, TT, RV). No complications were encountered throughout the study. In all patients, it was possible to place the planned implants six months after extraction. Generally, at the end of socket sealing surgery, a slight increase in vertical size was noted followed by a relative contraction of the same six months after the first surgery and at the end of implant placement surgery. At the end of the socket preservation procedure, the average increase in corono-apical size of sockets was 0.58 ± 0.43 mm (*p* < 0.001). After the six-month healing period the average increase in corono-apical size of the socket, as compared to pre-op dimension, was 0.27 ± 0.28 mm (*p* = 0.002). Therefore, there was a significant vertical contraction, respect to the post-preservation dimension, averaging 0.31 ± 0.24 mm (*p* < 0.001). Through superimposition of the pre-op and post-op models, applying the best-fit method, the mean volume variation in the treated sites was 0.026 ± 0.06 mm^3^ (*p* = 0.12). The volume analysis showed a palatal-lingual mean contraction of 0.33 ± 0.51 mm^3^ (*p* = 0.023) and a vestibular mean contraction of 0.8 ± 0.3 mm^3^ (*p* < 0.001). The difference was significant (*p* = 0.014). Finally, the analysis of width change based on sagittal section, both in the vestibular and palatal direction, showed general resorption, which was greater at the crestal level compared to more apical measurements. The mean contraction at the vestibular and palatal level, measured crestally, and at 3 mm and 7 mm apical respect to the crest, is shown in Table 1. At the palatal aspect the contraction at the crestal level, 3 mm and 7 mm apically, was respectively 1.84 ± 0.99 mm, 0.46 ± 0.23 mm and 0.31 ± 0.46 mm (Table 1).

## 4. Discussion

Tooth extraction leads to crestal bone volume reduction that varies from patient to patient but is considerable during the first three months after extraction 8, 9, 14, 15. Some studies showed that the vertical bone contraction is between 1.2 and 1.4 mm after six months [14,15]. Quantitative reductions in soft and hard tissues may have considerable clinical implications [16]. In fact, crestal volume loss may produce unsatisfactory aesthetic results [17], necessitating bone tissue augmentation at the time of implant placement. Most recent literature reviews agree that alveolar crest preservation is an efficient method of limiting the physiological reduction of the crestal volume loss following tooth extraction [18,19,20,21,22]. This study shows that a socket preservation technique that involves the insertion of deproteinized bovine bone into the socket, protected by porcine collagen matrix, is effective in maintaining the corono-apical size of the alveolar crest. Results show that after a six-month healing period prior to implant placement, an increase of about 0.27 mm in vertical socket size can be expected with respect to the time of tooth extraction. This outcome contradicts other results found in the literature. A recent randomized clinical study using a socket preservation technique, that consisted of a bone graft protected by a collagen matrix or a free epithelial/connective graft as compared to spontaneous healing, reported encouraging results [22]. Nevertheless, the vertical contraction after six months, calculated with CBCT, was 1.3 mm [22]. One possible explanation for the discrepancy of these results with those obtained in the present study may relate to the different methods used in measuring the crestal bone height during various follow-up sessions. In this study at the end of the socket seal stage, an average increase in the corono-apical size of 0.58 mm with a corresponding vertical contraction of only 0.31 mm at the end of the healing stage was observed. This clinical outcome suggests that collagen matrix provides effective protection of the graft used as socket seal. The best-fit method in pre- and post-op models (computerized superimposition of pre and post-op models) was shown to be effective given the high precision of superimposition of models. Hence, the measurements of volume variation can be considered reliable. Dimensional changes by superimposition technique are clinically relevant to determine if the volume is preserved or not [7]. The preservation of the volume is important from the prosthetic point of view, either functionally and esthetically, not only in implant dentistry, but even in traditional prosthetics [23]. The present results showed that socket seal protocol does not entirely prevent post-extraction alveolar crest remodeling. However, the volumetric analysis showed a small contraction both at the palatal (0.33 ± 0.51 mm^3^) and vestibular level (0.8 ± 0.3 mm^3^). Such remodeling did not jeopardize the placement of an implant in any of the 15 patients six months after the socket seal procedure. It may be hypothesized that these results are partly due to the flapless extraction technique, which causes less vascular trauma to periosteal blood vessels. Moreover, the use of a slow resorbing material like deproteinized bovine bone, and the protection of the same by a collagen matrix, ensured good stability of the graft. Finally, the analysis of linear contraction of the post-extraction socket at the crestal level and at 3 mm and 7 mm from crestal margin of the crest also showed how the resorption reduces as the distance from the crestal margin increases. Nevertheless, also the more apical zones can undergo a certain amount of resorption despite the regenerative protocol adopted, as shown in a previous study [22]. Furthermore regarding the histomorphometric measurements, as described in the previous report of the same dataset, residual xenograft particles (31.97% ± 3.52%) were surrounded by either newly formed bone (16.02% ± 7.06%) or connective tissue (50.67% ± 8.42%) without fibrous encapsulation. The collagen matrix underwent a physiological substitution process in favor of well-vascularized collagen-rich connective tissue [12].

## 5. Conclusions

The clinical protocol herein used for socket preservation, consisting of deproteinized bovine bone protected by a collagen matrix, is effective in contrasting the physiological remodeling process following tooth extraction. Further studies with wider sample size and longer follow-up are necessary to assess the long-term effects of this protocol.

## Figures and Tables

**Table 1:** Mean contraction (in mm) of the cortical plate at different levels, evaluated by 3D superimposition of scanned stone casts.

Case n	Palatal/Lingual Section			Vestib/Sagittal Section		
	crest	3 mm	7 mm	crest	3 mm	7 mm
1	1.6	0.11	0.22	1.68	1.27	0.41
2	0.89	0.48	0.00	1.04	0.68	0.65
3	4.02	1.00	0.35	1.85	0.54	0.95
4	3.24	0.43	0.08	2.74	0.89	0.25
5	0.93	0.41	0.26	4.07	2.92	0.46
6	1.28	0.63	1.79	1.78	0.88	0.69
7	2.54	0.26	0.8	1.89	0.87	1.08
8	2.11	0.38	0.14	1.48	0.93	0.91
9	1.72	0.12	0.03	1.83	1.01	0.64
10	2.46	0.48	0.41	2.57	1.81	1.04
11	1.69	0.24	0.00	1.52	0.68	0.33
12	0.41	0.71	0.15	1.92	1.25	0.86
13	0.73	0.61	0.06	1.79	1.41	0.99
14	2.61	0.58	0.18	2.48	1.78	1.18
15	1.41	0.43	0.21	1.73	1.58	1.35
mean	1.84	0.46	0.31	2.02	1.23	0.79
st.dev	0.99	0.23	0.46	0.71	0.61	0.33

The difference between palatal–lingual and vestibular side cortical plate width changes was not significant at crestal level (*p* = 0.57), while it was at 3mm (*p* < 0.001) and 7mm (*p* = 0.003) apically to the crest.

## Data Availability

The data presented in this study are available on request from the corresponding author.
